# Efficacies of radiotherapy in rectal cancer patients treated with total mesorectal excision or other types of surgery: an updated meta-analysis

**DOI:** 10.3389/or.2025.1567818

**Published:** 2025-05-01

**Authors:** Wenshu Wang, Runyuan Zhao, Xi Liang, Manjun Liu, Haiyan Bai, Jianli Ge, Binxi Yao, Zheng Zhi, Jianming He

**Affiliations:** ^1^ Department of Radiotherapy, Hebei Province Hospital of Chinese Medicine, Hebei University of Chinese Medicine, Shijiazhuang, China; ^2^ Department of Gastroenterology, Guang’anmen Hospital, China Academy of Chinese Medical Sciences, Beijing, China; ^3^ Key Laboratory of Integrated Chinese and Western Medicine for Gastroenterology Research (Hebei), Shijiazhuang, China

**Keywords:** rectal cancer, radiotherapy, concurrent chemoradiotherapy, surgery, TME

## Abstract

**Background:**

An updated meta-analysis was conducted to evaluate the efficacy of radiotherapy in rectal cancer patients treated with total mesorectal excision (TME) or other types of surgery (non-TME-only).

**Methods:**

The PubMed, Cochrane Library, and CNKI databases were searched. Data on overall survival (OS) were extracted.

**Results:**

Hazard ratios (HRs) for OS associated with preoperative radiotherapy, preoperative long-course concurrent chemoradiotherapy (LCCRT), preoperative radiotherapy alone, and postoperative radiotherapy in patients treated with TME were 1.02 [95% CI: 0.92–1.14, P = 0.65], 1.04 [95% CI: 0.93–1.16, P = 0.47], 0.87 [95% CI: 0.61–1.25, P = 0.46], and 1.18 [95% CI: 0.91–1.52, P = 0.20], respectively. HRs for OS associated with preoperative radiotherapy, preoperative LCCRT, preoperative radiotherapy alone, preoperative long-course RT (LCRT), and preoperative short-course radiotherapy (SCRT) in patients treated with non-TME-only surgery were 0.85 [95% CI: 0.79–0.90, P < 0.00001], 0.77 [95% CI: 0.63–0.94, P = 0.009], 0.86 [95% CI: 0.80–0.92, P < 0.0001], 0.83 [95% CI: 0.73–0.95, P = 0.005], and 0.84 [95% CI: 0.77–0.91, P= <0.0001], respectively. The HR for postoperative radiotherapy in patients treated with non-TME-only surgery was 1.08 [95% CI: 0.84–1.39, P = 0.57].

**Conclusion:**

Preoperative radiotherapy, regardless of the regimen, improves the OS in patients treated with non-TME-only surgery, but not in those treated with TME. Postoperative radiotherapy does not improve OS.

**Advances in knowledge:**

This meta-analysis will serve as a reference for decision-making in multidisciplinary approaches for rectal cancer patients.

## 1 Introduction

In 2022, an estimated 436,081 new cases of rectal cancer were reported in men and 293,621 in women, with 205,062 deaths in men and 138,699 in women (1). Its incidence is increasing in most developing countries, notably among young adults, and this may be partly attributed to lipid metabolism (1, 2, 3). Radiotherapy (RT) is widely accepted as an essential component of multidisciplinary treatment (MDT) for locally advanced rectal cancer (LARC), occurring in the mid and low rectum, primarily to reduce local recurrence (LR). However, MDT modalities vary considerably. Several RT regimens are available, such as concurrent chemoradiotherapy (CRT), sequential combinations of chemotherapy and RT (RT alone), long-course CRT (LCCRT), long-course RT (LCRT), and short-course RT (SCRT) (4, 5, 6).

Over the past decade, the standard treatment protocol for LARC consisted of neoadjuvant RT or CRT, followed by surgery and adjuvant systemic chemotherapy. Early response evaluation using diffusion-weighted magnetic resonance during neoadjuvant CRT has shown great promise in predicting the tumor response (7). Despite multiple efforts to potentiate preoperative CRT regimens, distant disease control and pathological complete response (pCR) remain suboptimal, with rates of approximately 25%–35% and 10%–15%, respectively (8, 9). To address the issues of distant metastasis and low pCR rates, total neoadjuvant therapy (TNT), which involves administering several cycles of chemotherapy either before RT/CRT (induction regimen) or after RT (consolidation regimen), has been tested and has shown promising results. TNT might improve metastasis-free survival, increase pCR and anal sphincter preservation rates, facilitate treatment adherence, and reduce toxicity (8, 10).

Recently, the supportive role of RT has been challenged. Total mesorectal excision (TME), the first-choice radical surgery, has been proposed as an alternative application to combined RT and surgery in patients with LARC, because TME significantly reduces LR without RT, thus questioning the necessity of RT either before or after surgery. van Gijn and Huh independently reported that the incidence of LR was less than 15% in the TME-alone group and preoperative RT did not improve overall survival (OS) (11, 12). Therefore, the impact of RT, particularly SCRT or postoperative RT, on OS remains a subject of debate. An updated meta-analysis was conducted to evaluate the efficacy of different RT regimens in patients with LARC treated with TME or other types of surgery (non-TME-only).

## 2 Materials and methods

### 2.1 Literature research

This study was designed in accordance with the Cochrane Handbook for Systematic Reviews of Interventions and the PRISMA (preferred reporting items for systematic reviews and meta-analyses) guidelines (3, 13). A systematic search of the PubMed, Cochrane Library, and CNKI databases was conducted to identify studies that examined the efficacy of RT in patients with rectal cancer. Articles written in either English or Chinese were included. The search strategy is detailed in the supplementary literature research.

### 2.2 Inclusion/exclusion criteria

The inclusion criteria were as follows: 1) patients: pathologically diagnosed rectal cancer at T1–T4N0–N + M0 stages. 2) Treatment: patients in the RT group underwent surgery followed by RT/CRT. Patients in the control group underwent surgery but did not receive RT/CRT. System therapy (chemotherapy, immunotherapy, target therapy, etc.) was not taken into account. 3) Study type: cohort. 4) Language: English or Chinese.

The exclusion criteria were as follows: 1) patients were treated with other forms of local treatment, including, but not limited to, radiofrequency ablation, cryoablation, high-intensity focused ultrasound, and others. 2) Duplicate published trials. 3) Studies without enough data.

### 2.3 Data extraction and quality assessment

Two authors independently retrieved and assessed the eligible articles. If there was any disagreement, the authors discussed and resolved the issue, with a third author adjudicating if the dispute could not be resolved. The following information was extracted: OS, cancer-specific survival, LR, local recurrence-free survival, disease-free survival (DFS), distant metastases-free survival (DMFS), and anal sphincter preservation rate. Some data, such as the OS curve, were extracted from images using the Engauge Digitizer software. The data were then independently cross-checked.

Risk of bias was assessed for the included cohort trials using the Cochrane Handbook 5.1.0. and Review Manager 5.3 (3, 13). Funnel plots were used to assess publication bias in the included studies.

### 2.4 Statistical analysis

Statistical analysis was performed using RevMan (Review Manager, version 5.3 for Windows). Statistical pooling of effect measures was based on the level of heterogeneity among studies, which was assessed using the Cochrane Q test and the I^2^ statistic. No significant heterogeneity was indicated by a P value >0.1 in the Cochrane Q test and an I^2^ statistic less than 50%. The prognostic effect was quantified using the hazard ratio (HR) and odds ratio (OR, with a 95% confidence interval (CI). The HR was calculated using the fixed-effects model with the inverse variance method. The OR was calculated using the Mantel-Haenszel method under the fixed-effects model. Publication bias was evaluated by visual inspection of funnel plots. A P value ≤0.05 was considered significant (3, 13).

## 3 Results

### 3.1 Characteristics of included studies

**FIGURE 1:**
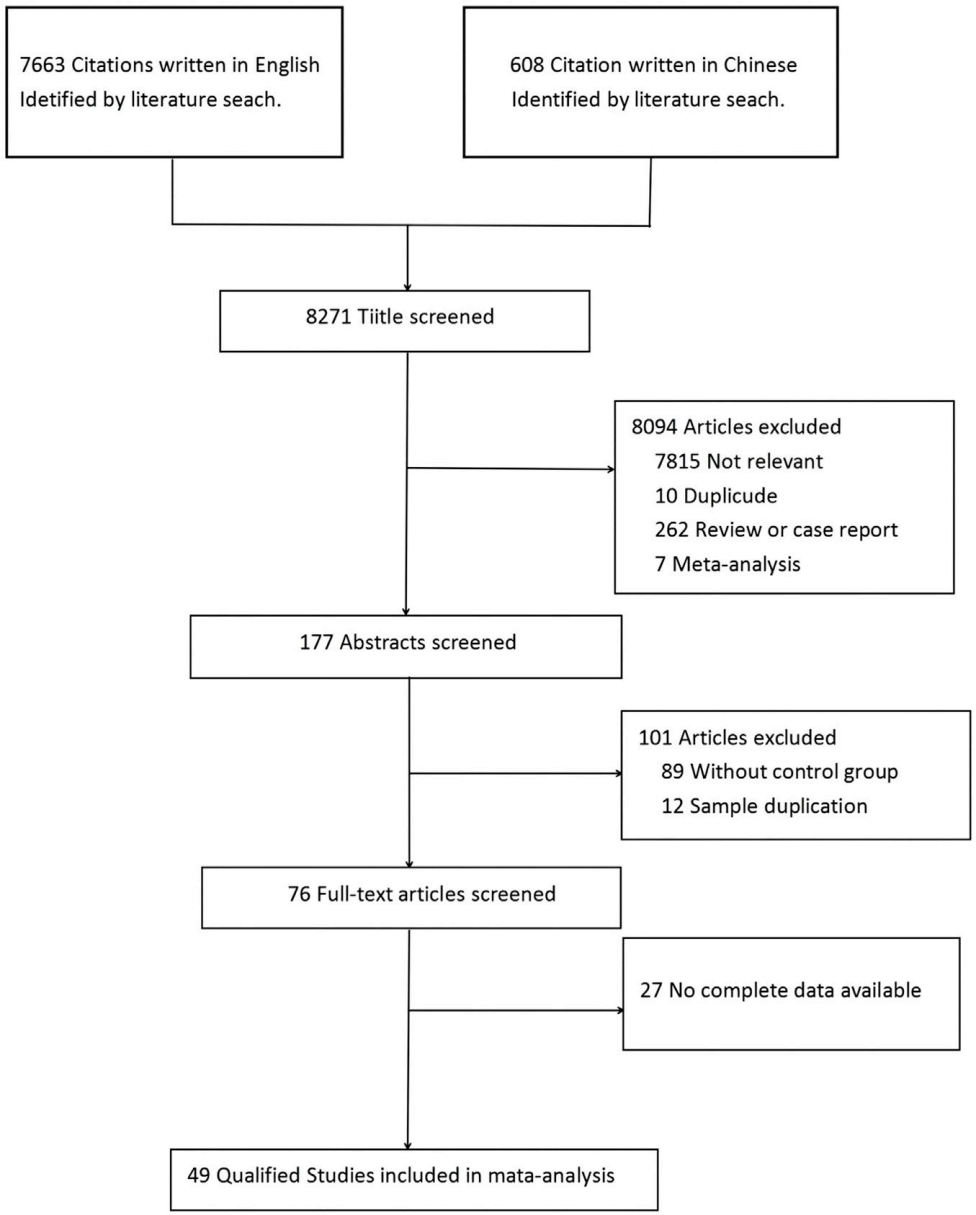
Meta-analysis literature search flow chart.

As shown in Figure 1, a total of 8,271 studies were identified initially using the above search strategy. After reviewing the titles and abstracts, 8,195 studies were excluded. After a thorough review of the full texts, 27 studies were excluded. Finally, 49 studies were eligible for meta-analysis (4, 5, 6, 11, 12, 14, 15, 16, 17, 18, 19, 20, 21, 22, 23, 24, 25, 26, 27, 28, 29, 30, 31, 32, 33, 34, 35, 36, 37, 38, 39, 40, 41, 42, 43, 44, 45, 46, 47, 48, 49, 50, 51, 52, 53, 54, 55, 56, and 57), including 26 retrospective studies (6, 21, 27, 28, 29, 30, 31, 32, 33, 34, 35, 36, 38, 39, 40, 41, 43, 45, 46, 48, 49, 51, 53, 55, 56, and 57) and 23 prospective studies (4, 5, 11, 12, 14, 15, 16, 17, 18, 19, 20, 22, 23, 24, 25, 26, 37, 42, 44, 47, 50, 52, and 54), with a total of 25,679 patients—13,278 in the RT group and 12,401 in the non-RT group (Figure 1). Table 1 lists the identified studies and their main characteristics.

**TABLE 1:** Characteristics of included studies.

Research type	Reference	Median follow-up time (months)	RT technique	Radiotherapy dose (Gy) Gy/day; total dose (Gy)	Group	Number of patients	T/N Stage	Median survival time (months)
Retro	(35)	156	NA	5 25	RT + Surgery	454	T1–T3	NA
					Surgery	454	T1–T3	NA
Retro	(41)	68 64	Linac	1.8 45	TME + CRT	309	T2/T3 N0–N2	NA
					TME + LPLD	176	T2/T3 N0–N2	NA
Retro	(28)	47.4	Linac	NA 40–45	CRT	48	T2–T4 N0–N2	NA
					CT	60	T2–T4 N0–N2	NA
Retro	(30)	24	NA	1.8–2.0 44–50.4	RTx	28	T1–T4 N0–N2	50
					Non-RTx	40	T1–T4 N0-N2	49
Retro	(55)	36	Linac	1.8 50.4	CCRT	37	T3–T4 N0-N2	NA
					Surgery	86	T3–T4 N0–N2	NA
Retro	(27)	NA	Linac	1.8–2.0/1.8–2.3 27-30/44.6–52	nRTx	28	T1–T4	42
					No-nRTx	65	T1–T4	38
Retro	(43)	29/27	NA	1.8–2.0 45–50.4	RTX	40	T1-T4 N0–N2	27 ± 4.81
					NRTX	46	T1–T4 N0–N2	24 ± 6.76
Retro	(39)	NA	NA	NA	CRT	3022	T1–T3	NA
					None	1,354	T1–T3	NA
Retro	(34)	45.7 ± 19.8	NA	NA 45–50	PCRT + surgery	70	NA N0–N2	NA
					Surgery	70	NA N0–N2	NA
Retro	(36)	43	NA	NA 30-55.8/20-25/10-12.5	RT	47	T3–T4 N0–N2	NA
					No-RT	46	T3–T4 N0–N2	NA
Retro	(40)	58	NA	NA 50.4	CRG	253	T2–T3	NA
					CG	460	T2–T3	NA
Retro	(53)	71	NA	NA 50	CCRT + TME	90	T3–T4 N0–N2	NA
					TME	94	T3–T4 N0–N2	NA
Retro	(38)	55.8	NA	NA	S + RT	386	T1–T4	NA
					S	635	T1–T4	NA
Retro	(49)	56.4 57.1	3DCRT	2 46–55	CRT	115	T3	NA
					CT	150	T3	NA
Retro	(21)	52	NA	5 25	RT + Surgery	94	T3 N1–N2	NA
					Surgery	57	T3 N1–N2	NA
Retro	(56)	48.1	NA	NA 25–50.4	CRT	102	T2–T3 N0–N2	NA
					CT	161	T2–T3 N0–N2	NA
Retro	(6)	41.5 43.1	NA	1.8–2.0 40–50.4	CRT	189	T2–T3 N1–N3	NA
					Surgery	649	T2–T3 N1–N3	NA
Retro	(29)	71	NA	1.8–2.0 43.2–60	CCRT	143	T3	NA
					Surgery	122	T3	NA
Retro	(48)	65.7 ± 29	NA	1.8–2.0 45–50.4	PCRT	1,258	T1–T3 N0–N2	NA
					Non-PCRT	957	T1–T3 N0–N2	NA
Retro	(51)	45	3DCRT	1.8–2.0 45–50.4	NACRT	185	T2–T3	NA
					Non-NACRT	173	T2–T3	NA
Retro	(46)	26	NA	2 50	NRCT	55	T3–T4 N0–N2	NA
					NCT	22	T3–T4 N0–N2	NA
Retro	(45)	46	NA	1.8–2.0 46–50.4	CRT	50	T2–T3 N0–N+	NA
					CT	31	T2–T3 N0–N+	NA
Retro	(54)	47.5	NA	40–50	NCRT	76	T3–T4 N0–N+	NA
					NCT	52	T3–T4 N0–N+	NA
Retro	(32)	60	NA	NA	CRT	1,384	T1–T3	NA
					CT	718	T1–T3	NA
Retro	(33)	33.9	NA	NA	NCRT	80	T2–T3 N0–N2	NA
					NCT	238	T2–T3 N0–N2	NA
Retro	(31)	NA	NA	NA	EP + CCRT	107	T1 N0–N3	NA
					EP	718	T1 N0–N3	NA
Retro	(57)	NA	IMRT/VMRT	NA 45–66.4	RT	69	T2/T3 N0–N2	NA
					Non-RT	294	T2/T3 N0–N2	NA
RCT	(24)	NA	NA	NA 20	Multiple fractions	272	NA	NA
					No-xRT	275	NA	NA
RCT	(16)	NA	Linac	2.3 34.5	RT + Surgery	152	T2–T4	NA
					Surgery	166	T2–T4	NA
RCT	(25)	80	NA	NA 40–44	CCRT	46	NA	NA
					Control	58	NA	NA
RCT	(37)	75	Linac	2.3 34.5	RT	166	T2–T4	NA
					Non-RT	175	T2–T4	NA
RCT	(4)	53	Linac	5 25	RT	424	T1–T3	NA
					Non-RT	425	T1–T3	NA
RCT	(15)	54	Linac	1.75 31.5	RT	159	NA	NA
					Non-RT	150	NA	NA
RCT	(17)	NA	NA	5 45–60	RT + surgery	228	Dukes A-C2	NA
					Surgery	239	Dukes A-C2	NA
RCT	(47)	NA	Linac	5 20	RT	143	NA	NA
					Non-RT	141	NA	NA
RCT	(26)	50	Linac	5 25	RT	272	NA	NA
					Non-RT	285	NA	NA
RCT	(5)	120	Linac	2.0 40	RT	139	NA	31
					Non-RT	140	NA	24
RCT	(22)	NA	Three-field technique	2.0 46	RT	66	Dukes B andC	NA
					Non-RT	70	Dukes B and C	NA
RCT	(20)	63	Box of three-field	1.8 38	RT + IORT	69	T1-T3	NA
					TME	44	T1-T3	NA
RCT	(18)	34	NA	18–20	IORT	19	T1-T3	NA
					Control	22	T1-T3	NA
RCT	(44)	84	NA	5 25	RT	379	T1-T4 N0-N2	NA
					Non-RT	376	T1-T4 N0-N2	NA
RCT	(42)	78	Linac	1.8 50.4–54	CRT	122	NA	NA
					CT	29	NA	NA
RCT	(12)	52	NA	1.8 45–50.4	CRT	64	T3 N1–N2	NA
					CT	190	T3 N1–N2	NA
RCT	(11)	144	NA	5 25	RT + TME	897	T0–T4	139.2
					TME	908	T0–T4	139.2
RCT	(50)	30	3D-EBRT	1.8–2.0 44–45	RT	206	T3–T4	NA
					Non-RT	178	T3–T4	NA
RCT	(23)	44	3DCRT	2 50	CRT	42	T3	NA
					CT	99	T3	NA
RCT	(19)	36	3DCRT	2 50	preCRT	25	T1–T4 N0–N2	NA
					Non-preCRT	16	T1–T4 N0–N2	NA
RCT	(52)	55	NA	1.8 50.4	nCRT	395	T3–T4 N0–N+	NA
					Surgery	395	T3–T4 N0–N+	NA
RCT	(14)	NA	NA	1.8–2.0 50.4–54	RT	174	T2–T3 N0–N2	NA
					Non-RT	92	T2–T3 N0–N2	NA

In all included studies, their baselines were comparable. The risk of bias assessment is shown in the supplementary material (Supplementary Figure 1, 2). None of the studies were at high risk of bias.

#### 3.1.1 RT improves sphincter preservation and reduces LR

**FIGURE 2:**
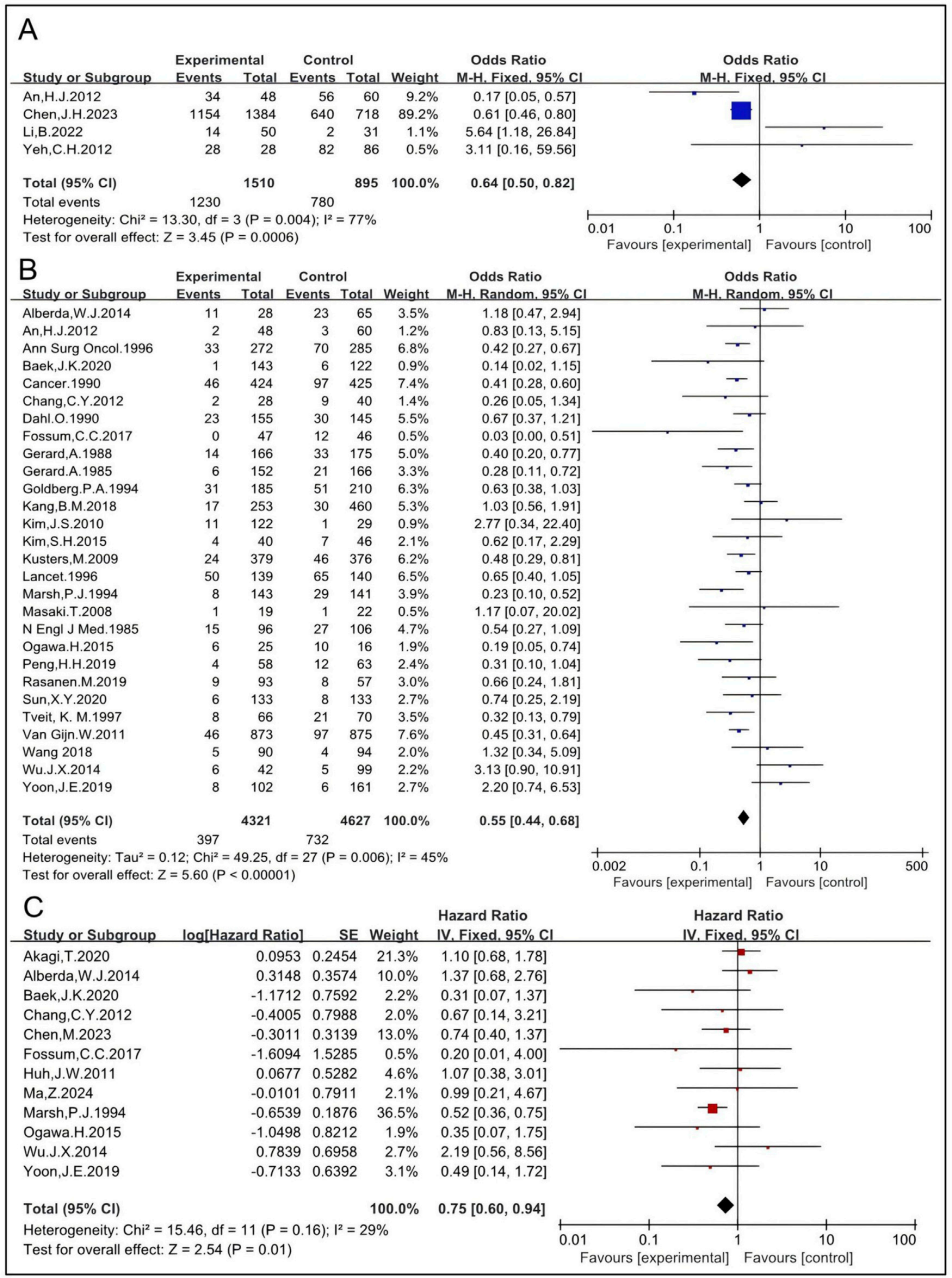
OR for anal sphincter preservation rate (A). OR for LR rate **(B)**. HR for LR-free survival rate **(C)**.

RT, as a local treatment to surgical resection, has been used in select cases to avoid permanent colostomy and reduce LR (45). Only four studies provided enough data to analyze the OR for sphincter preservation rate (28, 32, 45, 55). A total of 2,405 subjects were included, with 1,510 in the RT group and 895 in the non-RT group. The pooled OR was 0.64 [95% confidence interval (CI): 0.50–0.82, P = 0.0006] (Figure 2), indicating that RT significantly increases the sphincter preservation rate.

A total of 28 studies, including 4,321 patients in the RT group and 4,627 in the non-RT group, were eligible to analyze the LR rate (4, 5, 11, 15, 16, 17, 18, 19, 21, 22, 23, 25, 26, 27, 28, 29, 30, 36, 37, 40, 42, 43, 44, 47, 49, 51, 53, and 56). The pooled OR was 0.55 [95% CI: 0.44–0.68, P < 0.00001] (Figure 2), indicating that the LR rate in the RT group is significantly lower than that in the non-RT group. Twelve studies reported LR-free survival rate, involving 3,205 subjects—1,116 in the RT group and 2,089 in the non-RT group (6, 12, 19, 23, 27, 29, 30, 33, 36, 47, 56, 57). The pooled OR was 0.75 [95% CI: 0.60–0.94, P = 0.01] (Figure 2), indicating that the LR-free survival rate in the RT group is significantly higher than that in the non-RT group. The I^2^ was 45% and RT modalities may be involved in the level of heterogeneity, which has been analyzed below. Therefore, the results should be interpreted with caution.

#### 3.1.2 Preoperative RT improves LR of patients treated with TME while postoperative RT may not

TME is one of the first-choice surgical treatments for middle and low LARC because it significantly reduces LR, thus questioning the necessity of RT. To address this question, the impact of RT before and after TME on LR was analyzed.

**FIGURE 3:**
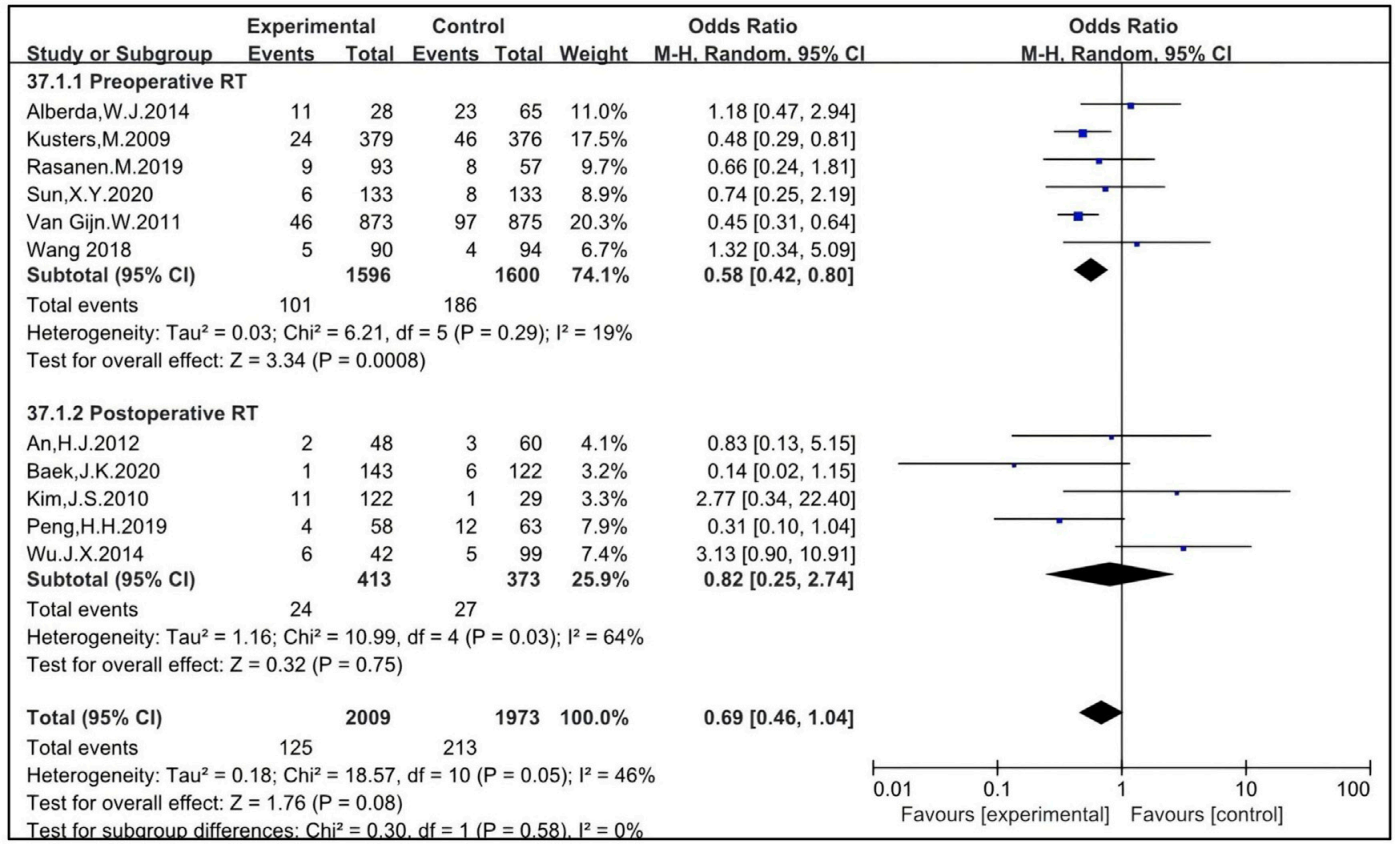
Impact of preoperative and postoperative RT on LR of patients treated with TME.

Six studies were eligible to analyze the impact of preoperative RT, with 1,596 patients enrolled in the RT group and 1,600 in the non-RT group (11, 21, 27, 44, 51, 53). The OR was 0.58 [95% CI: 0.42–0.80, P = 0.0008]. Five studies were eligible to analyze the impact of postoperative RT, with 413 patients enrolled in the RT group and 373 in the non-RT group (23, 28, 29, 42, 49). The OR was 0.82 [95% CI: 0.25–2.74, P = 0.75]. The pooled OR for LR with the combination of preoperative and postoperative RT was 0.69 [95% CI: 0.46–1.04, P = 0.08] (Figure 3). This indicates that preoperative RT improves LR in patients treated with TME while postoperative RT may not.

#### 3.1.3 Preoperative RT improves LR of patients treated with non-TME-only surgery

**FIGURE 4:**
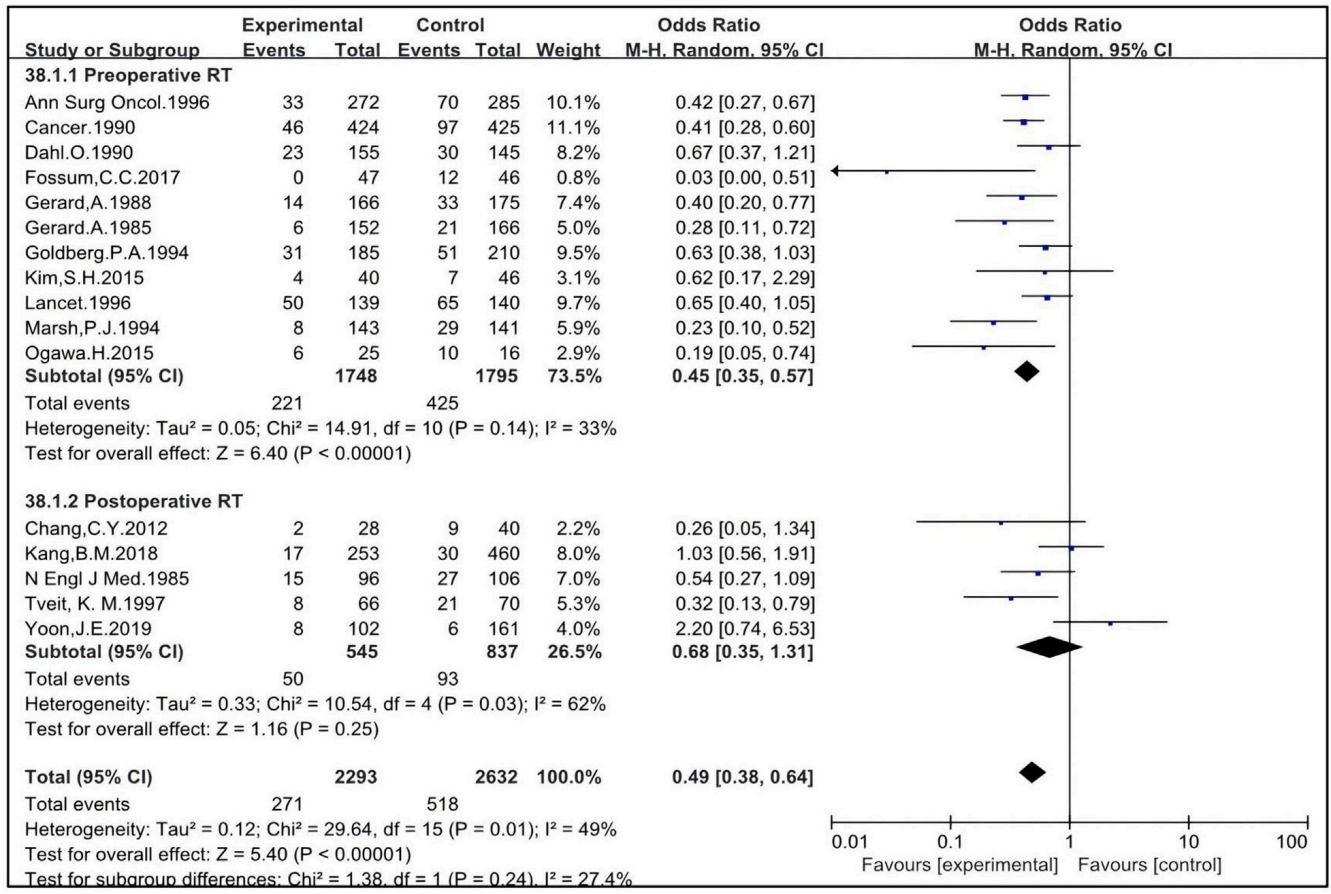
Impact of preoperative and postoperative RT on LR of patients treated with non-TME.

Sixteen studies included patients who underwent surgical procedures other than TME or not clearly defined as “radical surgery”. These studies were eligible to analyze the impact of preoperative and postoperative RT on LR in patients treated with non-TME surgeries. Eleven studies were eligible to analyze the impact of preoperative RT, with 1,748 patients enrolled in the RT group and 1,795 in the non-RT group (4, 5, 15, 16, 17, 19, 26, 36, 37, 43, 47). The OR was 0.45 [95% CI: 0.35–0.57, P < 0.00001]. Five studies were eligible to analyze the impact of postoperative RT, with 545 patients enrolled in the RT group and 837 in the non-RT group (22, 25, 30, 40, 56). The OR was 0.68 [95% CI: 0.35–1.31, P = 0.25]. The pooled OR for LR with the combination of preoperative and postoperative RT was 0.49 [95% CI: 0.38–0.64, P < 0.00001] (Figure 4). Preoperative RT improves the LR of patients treated with non-TME-only surgery.

#### 3.1.4 RT improves survival

**FIGURE 5:**
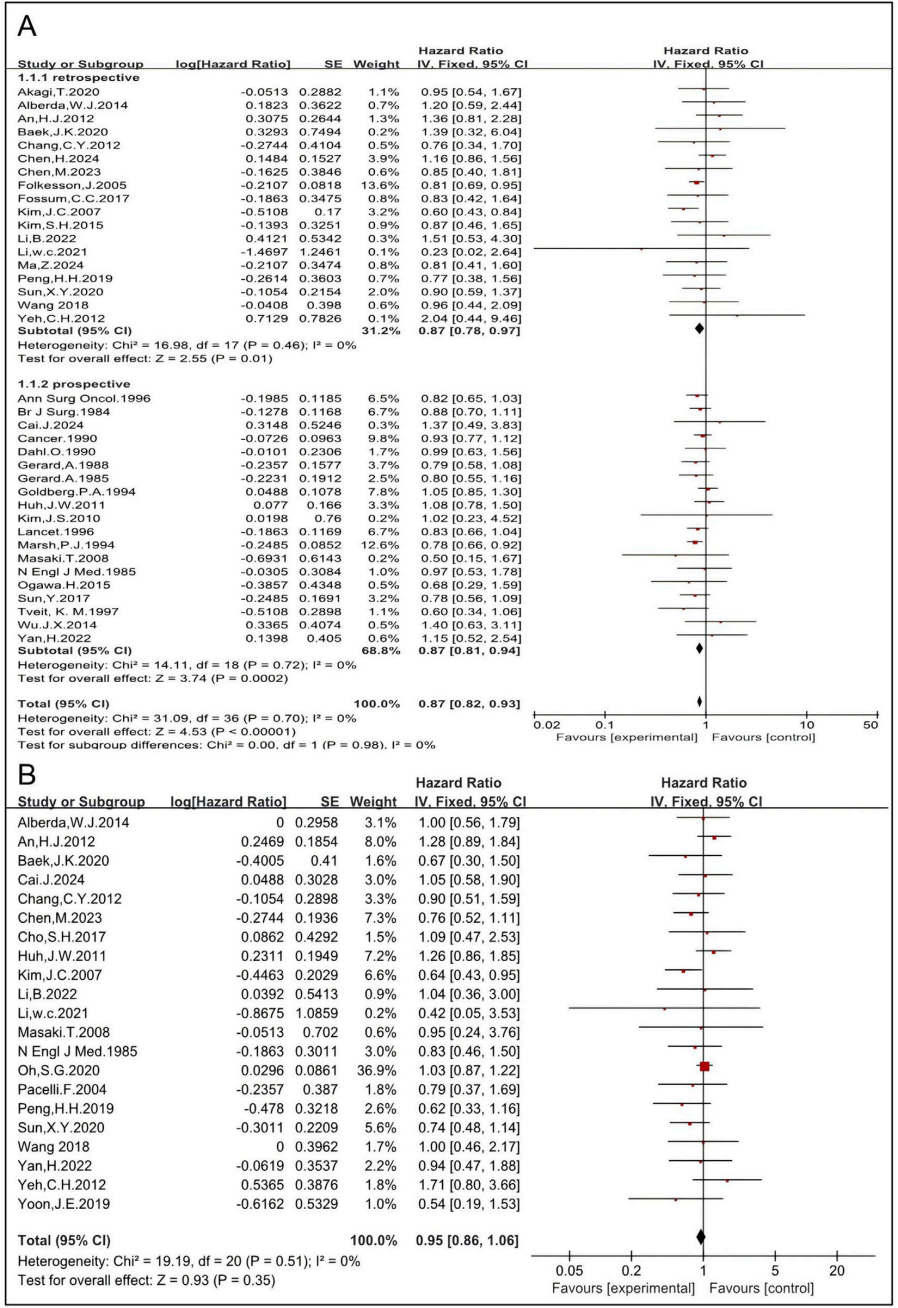
Pooled HRs for the survival rate in patients treated with RT **(A)**. Pooled HRs for DFS in patients treated with RT **(B)**.

Thirty-nine studies, including 18 retrospective and 19 prospective studies, were eligible to analyze the impact of preoperative and postoperative RT on OS in rectal cancer patients. The HRs for OS in the retrospective (6, 27, 28, 29, 30, 31, 33, 35, 36, 41, 43, 45, 46, 49, 51, 53, 55, 57) and the prospective (4, 5, 12, 14, 15, 16, 17, 18, 19, 22, 23, 24, 25, 26, 37, 42, 47, 52, 54) studies were 0.87 [95% CI: 0.78–0.97, P = 0.01] and 0.87 [95% CI: 0.81–0.94, P = 0.0002], respectively. The pooled HR of both retrospective and prospective studies was 0.87 [95%CI: 0.82–0.93, p = 0.0002] (Figure 5), indicating that RT significantly improves OS. Three studies reported cancer-specific survival (32, 44, 50). The pooled HR was 0.78 [95%CI: 0.68–0.90, P = 0.0005] (Supplementary Figure S3), indicating that RT improves cancer-specific survival. The HRs for OS and cancer-specific survival concordantly support that RT improves survival. The DFS was analyzed and 21 studies were eligible (12, 14, 18, 20, 25, 27, 28, 29, 30, 33, 34, 41, 45, 46, 48, 49, 51, 53, 54, 55, 56). The pooled HR, including 3,037 patients in the RT group and 2,903 in the non-RT group, was 0.95 [95% CI: 0.86–1.06, P = 0.35] (Figure 5), indicating that there is no significant difference in DFS between the RT group and the non-RT group. The metastasis-free survival was also analyzed and six studies were eligible (19, 27, 33, 36, 47, 57). The pooled HR, including 489 patients in the RT group and 887 in the non-RT group, was 1.02 [95% CI: 0.84–1.24, P = 0.86] (Supplementary Figure S4), indicating there is no significant difference in metastasis-free survival between the RT group and the non-RT group. Taken together, these results imply that RT may not improve DFS or metastasis-free survival.

CRT yields better results than RT alone in terms of survival outcomes in certain cancer types. The impact of CRT on OS was analyzed. Thirty-eight studies, of which 26 studies (6, 12, 14, 19, 22, 23, 25, 27, 28, 29, 30, 31, 33, 36, 41, 42, 43, 45, 46, 49, 50, 51, 52, 53, 54, 55) employed CRT and 11 (4, 5, 15, 16, 17, 18, 24, 26, 35, 37, 47) employed RT-alone, were eligible. The HRs of CRT and RT alone were 0.91 [95% CI: 0.82–1.02, p = 0.10] and 0.85 [95% CI: 0.80–0.92, P < 0.00001]. The pooled HR, including 2,867 patients in the RT group and 3,895 in the non-RT group, was 0.87 [95% CI: 0.82–0.92, P < 0.00001] (Supplementary Figure S5). Further analysis was conducted understand these results.

#### 3.1.5 Preoperative RT improves OS while postoperative RT might not

**FIGURE 6:**
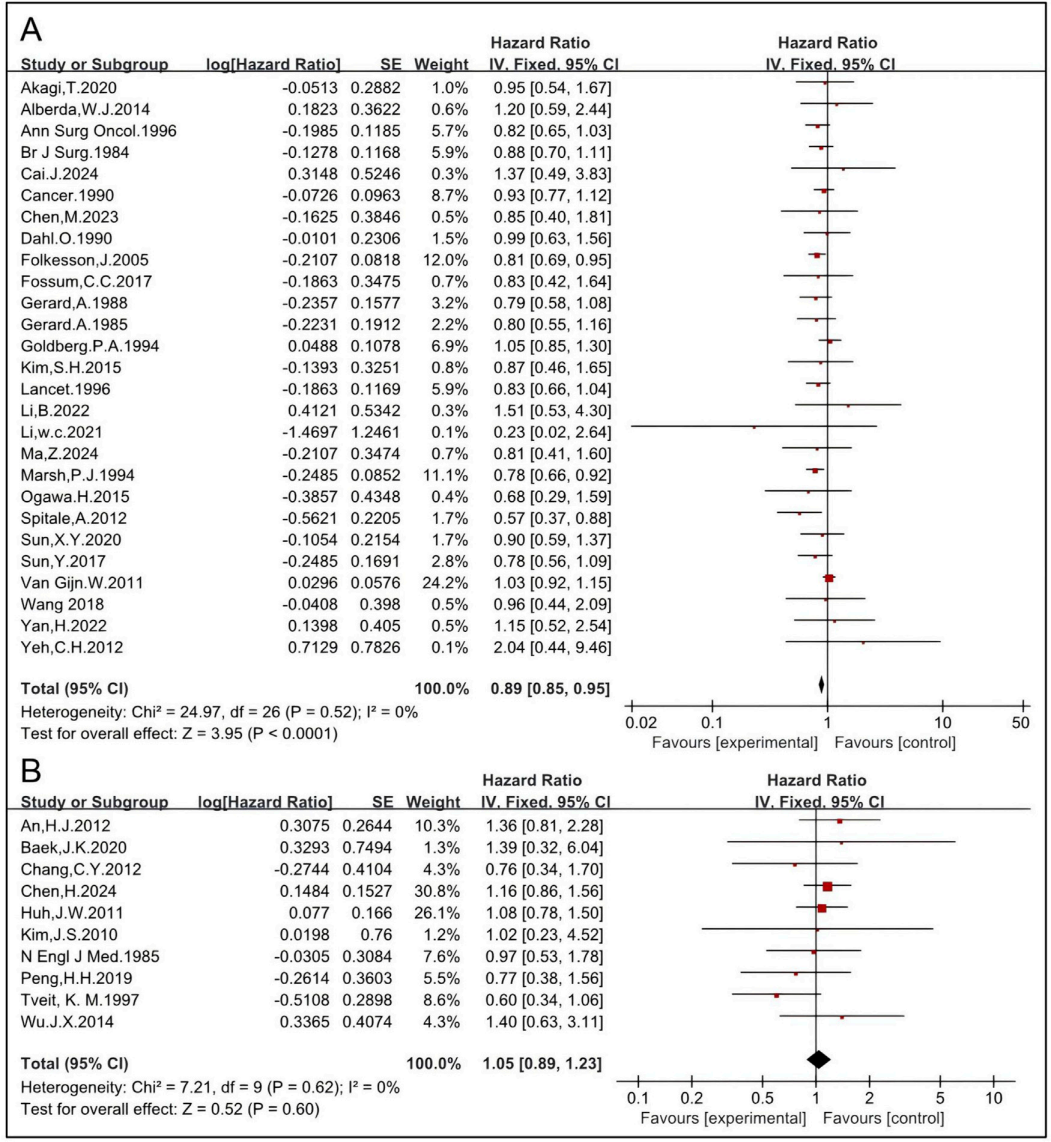
Impact of preoperative RT on OS **(A)**. Impact of postoperative RT on OS **(B)**.

Although there has been a paradigm shift from a postoperative to a preoperative approach, few randomized studies have directly compared preoperative RT with postoperative RT (58, 59). Here, the effects of preoperative and postoperative RT on OS were analyzed. The pooled HR of preoperative RT of 27 studies, including 5,152 patients in the RT group and 5,835 in the non-RT group, was 0.89 [95% CI: 0.85–0.95, P < 0.0001] (4, 5, 6, 11, 14, 15, 16, 17, 19, 24, 26, 27, 33, 35, 36, 37, 43, 45, 46, 47, 50, 51, 52, 53, 54, 55, 57) (Figure 6). The pooled HR of postoperative RT of 10 studies, including 781 patients in the RT group and 1,536 in the non-RT group, was 1.05 [95% CI: 0.89–1.23, P = 0.60] (12, 22, 23, 25, 28, 29, 30, 31, 42, 49) (Figure 6), indicating that preoperative RT significantly improves OS while postoperative RT might not.

**FIGURE 7:**
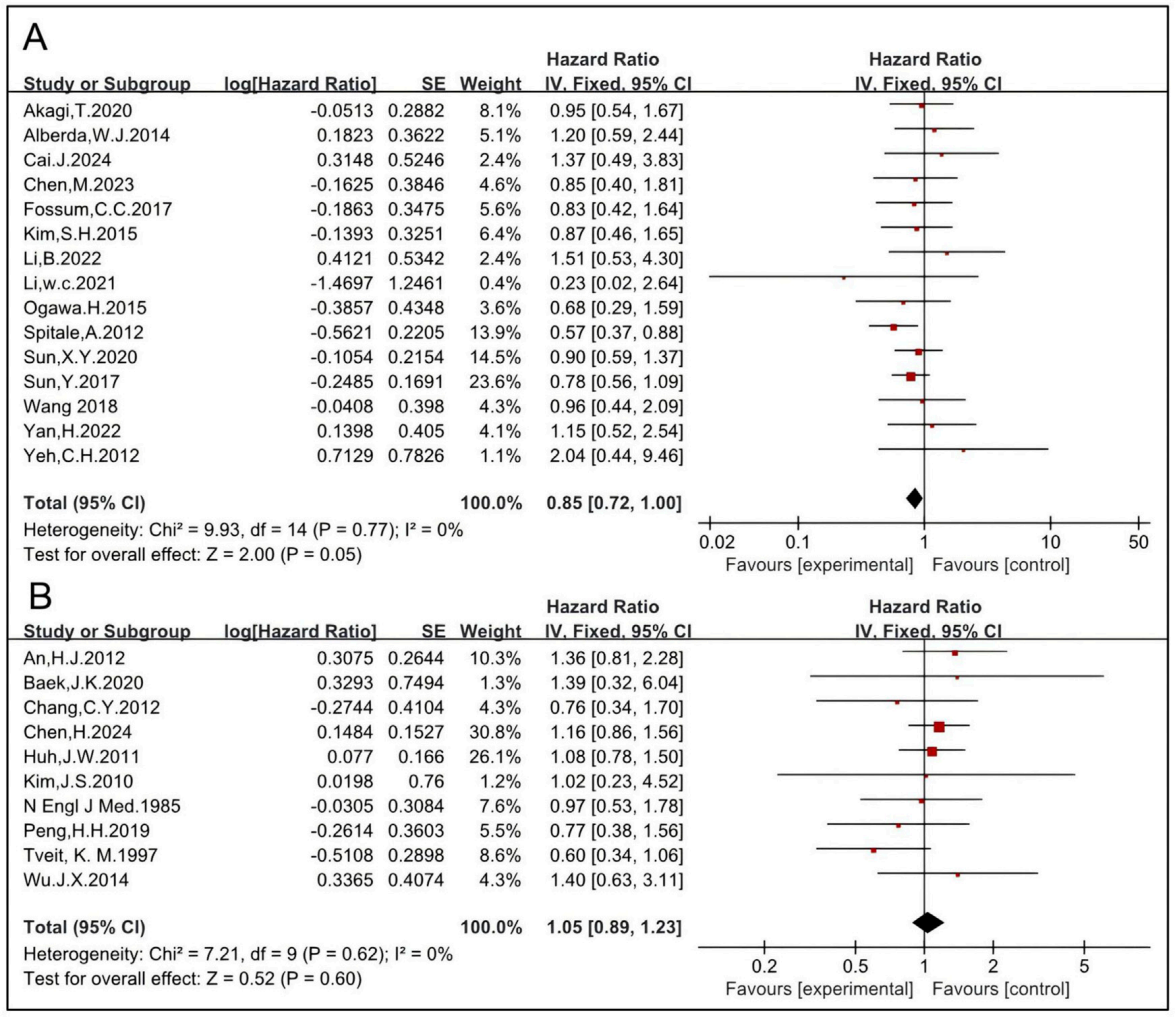
Impact of preoperative CRT on OS **(A)**. Impact of postoperative CRT on OS **(B)**.

The effects of CRT before and after surgery on OS were analyzed. The pooled HR of preoperative CRT of 15 studies, including 1,777 patients in the RT group and 2,183 in the non-RT group, was 0.85 [95% CI: 0.72–1.00, P = 0.05] (6, 14, 19, 27, 33, 36, 43, 45, 46, 50, 51, 52, 53, 54, 55) (Figure 7). The pooled HR of postoperative CRT of 10 studies, including 781 patients in the RT group and 1,536 in the non-RT group, was 1.05 [95% CI: 0.89–1.23, P = 0.60] (12, 22, 23, 25, 28, 29, 30, 31, 42, 49) (Figure 7), indicating that preoperative CRT significantly improves OS while postoperative CRT might not. These results are concordant with those of RT and indicate that preoperative RT/CRT significantly improves OS while postoperative RT/CRT might not.

**FIGURE 8:**
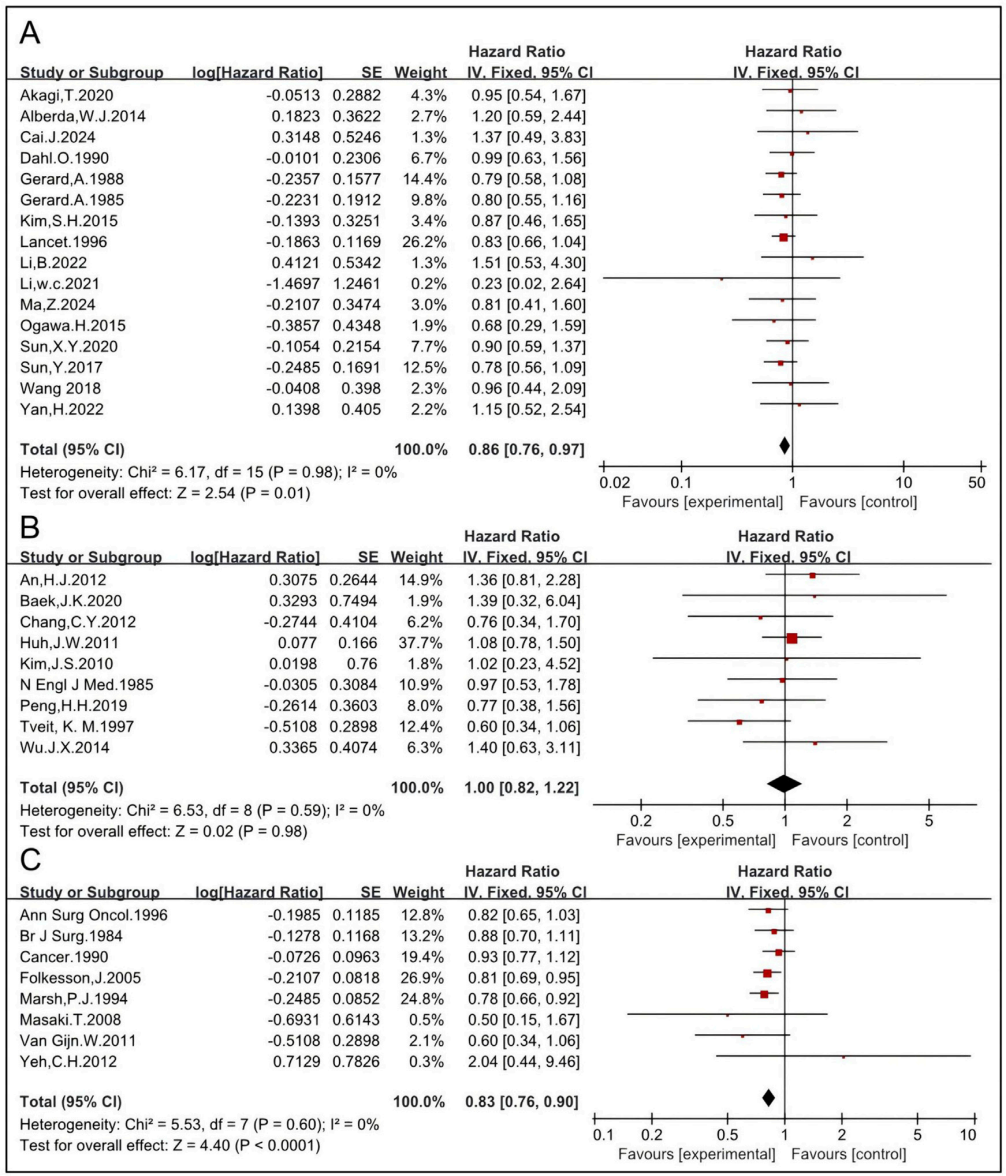
Impact of preoperative LCRT on OS **(A)**. Impact of postoperative LCRT on OS **(B)**. Impact of SCRT on OS **(C)**.

The impact of different RT regimens on OS was analyzed. LCRT typically involves a total dose of approximately 45–50 Gy delivered in 1.8–2 Gy fractions over a period of 5–6 weeks, whereas SCRT consists of a total dose of approximately 25 Gy delivered in 5 Gy fractions over the course of 1 week. Sixteen studies, including 2,092 patients in the RT group and 2,560 in the non-RT group, were eligible to analyze the impact of preoperative LCRT. The pooled HR was 0.86 [95% CI: 0.76–0.97, P = 0.01] (5, 6, 14, 15, 16, 19, 27, 37, 43, 45, 46, 51, 52, 53, 54, 57) (Figure 8), indicating that preoperative LCRT significantly improves OS. Nine studies, including 674 patients in the RT group and 818 in the non-RT group, were eligible to analyze the impact of postoperative LCRT. The pooled HR was 1.00 [95% CI: 0.82–1.22, P = 0.98] (12, 22, 23, 25, 28, 29, 30, 42, 49) (Figure 8), which is concordant with the results observed for postoperative RT mentioned above. SCRT was used without chemotherapy before surgical resection and eight studies, including 2,518 patients in the RT group and 2,596 in the non-RT group, were eligible (4, 11, 18, 24, 26, 35, 47, 55). The HR was 0.83 [95% CI: 0.76–0.90, P < 0.0001] (Figure 8). Taken together, these results indicate that both preoperative LCRT and preoperative SCRT significantly improve OS while postoperative LCRT might not.

#### 3.1.6 RT may not improve OS of patients treated with TME

**FIGURE 9:**
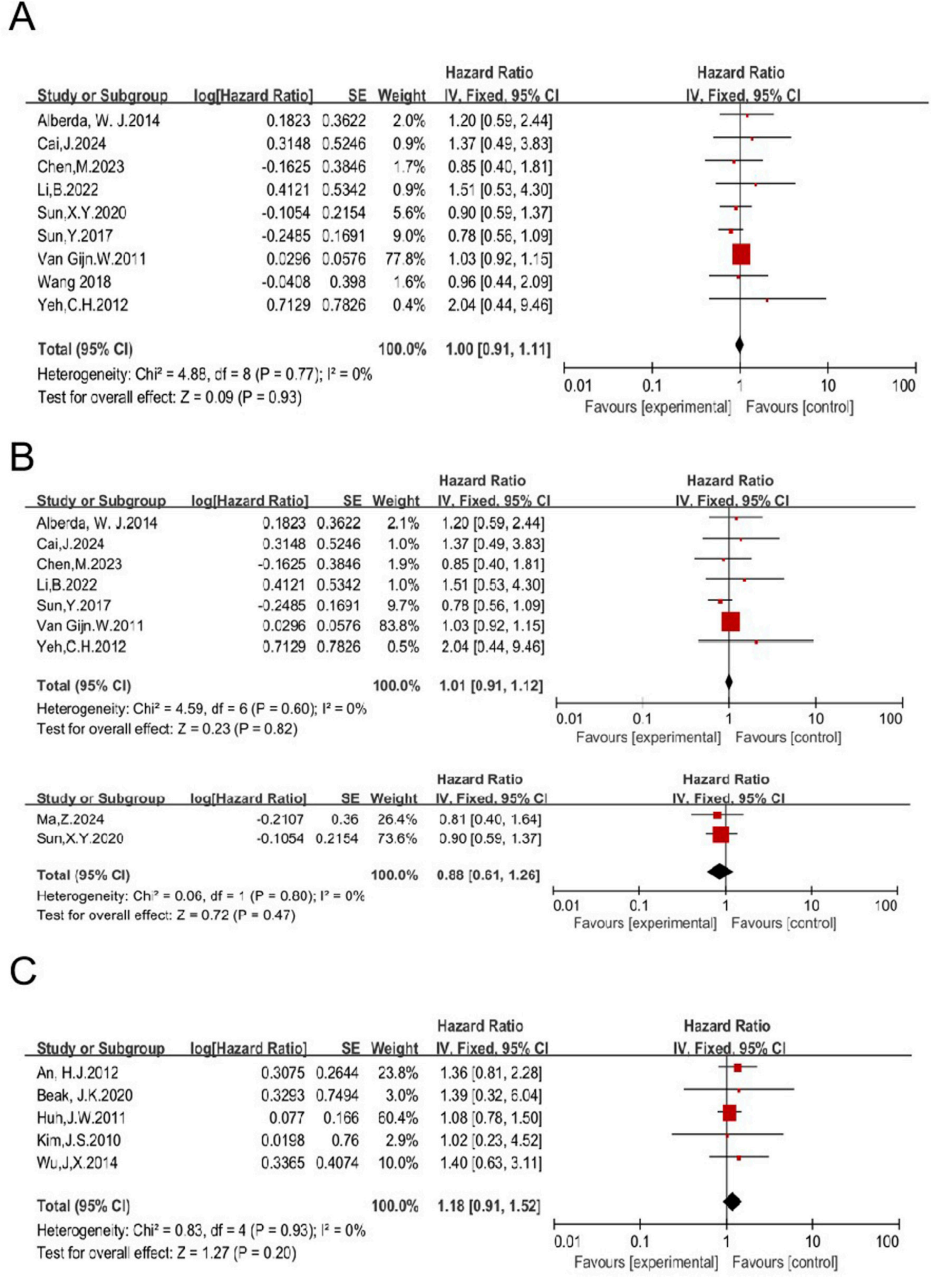
Impact of preoperative RT on OS of patients treated with TME **(A)**. Impacts of preoperative CRT and preoperative RT-alone on OS in patients treated with TME **(B)**. Impact of postoperative RT on OS in patients treated with TME **(C)**.

The impact of RT on OS in patients treated with TME was analyzed. Nineteen studies compared OS of patients receiving a combination of RT and TME vs. TME alone, and the pooled HR of was 1.02 [95% CI: 0.94–1.12, P = 0.59] (11, 12, 14, 18, 23, 27, 28, 29, 33, 41, 42, 45, 46, 49, 51, 52, 53, 55, 57) (Supplementary Figure S6). Nine studies, including 1,610 patients enrolled in the RT group and 1,981 in the non-RT group, were eligible to analyze the impact of preoperative RT (11, 14, 27, 33, 45, 51, 53, 55, 57). The HR was 1.00 [95% CI: 0.91–1.11, P = 0.93] (Figure 9). Preoperative LCCRT was employed in seven of nine studies. A total of 942 patients were enrolled in the RT group, while 1,088 patients were included in the non-RT group (11, 14, 27, 33, 45, 52, 55). The HR was 1.01 [95% CI: 0.91–1.12, P = 0.82]. Preoperative RT-alone was employed in two of nine studies. A total of 254 patients were enrolled in the RT group, while 467 patients were included in the non-RT group (51, 57). The HR was 0.88 [95% CI: 0.61–1.26, P = 0.47] (Figure 9). These results concordantly indicate that preoperative RT, regardless of the regimen used (including LCCRT or RT alone), may not improve OS of patients treated with TME.

Five studies were eligible to analyze the impact of postoperative RT on OS, with 454 patients enrolled in the RT group and 532 in the non-RT group (12, 23, 28, 29, 42). The HR was 1.18 [95% CI: 0.91–1.52, P = 0.20] (Figure 9). Taken together with the aforementioned data, these results concordantly indicate that RT, regardless of the regimen, may not improve OS of patients treated with TME, suggesting that RT may not be necessary.

#### 3.1.7 Preoperative RT improves OS of patients treated with non-TME-only surgery

**FIGURE 10:**
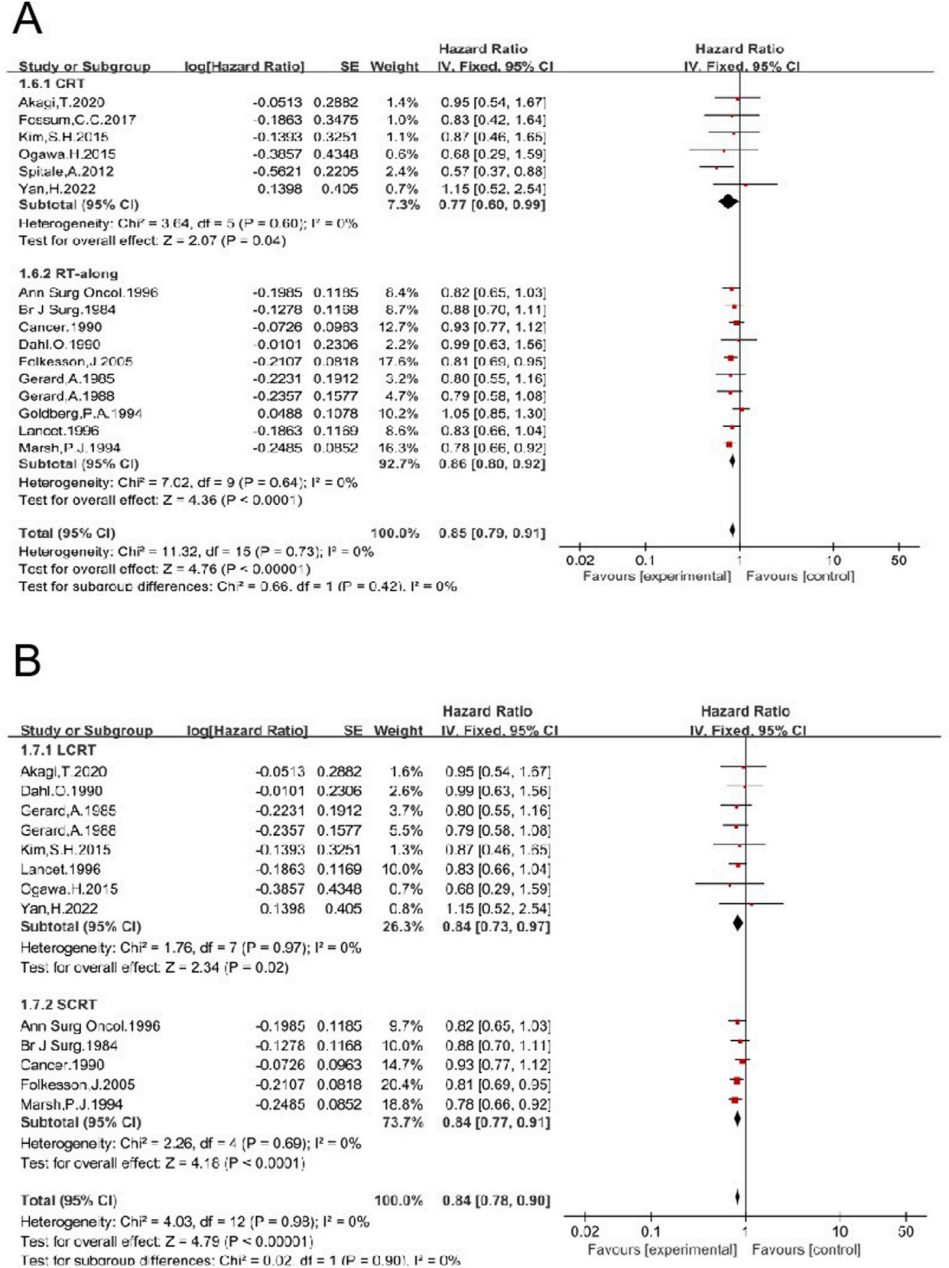
Impacts of preoperative CRT and preoperative RT-alone on OS in patients treated with non-TME sugery **(A)**. Impacts of preoperative LCRT and preoperative SCRT on OS in patients treated with non-TME sugery **(B)**.

Nineteen studies enrolled 6,525 patients who underwent surgical treatment other than TME (non-TME-only). OS was compared between patients who received a combination of RT and non-TME-only surgery vs. those who underwent non-TME-only surgery alone (4, 5, 6, 15, 16, 17, 19, 24, 25, 26, 30, 31, 35, 36, 37, 43, 47, 50, 54). The pooled HR was 0.86 [95% CI: 0.81–0.92, P < 0.00001] (Supplementary Figure S7), indicating that RT significantly improves OS. Preoperative RT was employed in 16 trials, including 3,542 patients in the RT group and 3,854 in the non-RT group (4, 5, 6, 15, 16, 17, 19, 24, 26, 35, 36, 37, 46, 47, 50, 54). The HR was 0.85 [95% CI: 0.79–0.91, P < 0.00001]. Six studies were eligible to analyze the impact of preoperative LCCRT on OS, and the HR was 0.77 [95% CI: 0.60–0.99, P = 0.04] (6, 19, 36, 43, 50, 54). Ten studies analyzed the effect of preoperative RT-alone on OS, and the HR was 0.86 [95% CI: 0.80–0.92, P < 0.0001] (4, 5, 15, 16, 17, 24, 26, 35, 37, 47) (Figure 10). Preoperative LCRT was employed in eight trials, and the HR was 0.84 [95% CI: 0.73–0.97, P = 0.02] (5, 6, 15, 16, 19, 37, 43, 54). Preoperative SCRT was employed in five trials, and the HR was 0.84 [95% CI: 0.77–0.91, P = <0.0001] (4, 24, 26, 35, 47) (Figure 10). These results concordantly indicate that each preoperative RT regimen, including LCCRT, RT-alone, LCRT, and SCRT, significantly improves OS of patients treated with non-TME-only surgery.

Postoperative RT was employed in three trials, including 181 patients in the RT group and 816 in the non-RT group (25, 30, 31). The HR was 1.08 [95% CI: 0.84–1.39, P = 0.57] (Supplementary Figure S7), indicating that postoperative RT may not improve OS of patients treated with non-TME-only surgery.

## 4 Discussion

The management of LARC requires a multidisciplinary approach, with treatment specific to each patient based on a thorough assessment of disease risks. Multimodality treatment, along with the optimization of individual treatment components, have contributed to and improved prognosis (14, 31, 33, 57). At present, several MDT modalities are employed for LARC, with surgery playing a pivotal role. TME, combined with either preoperative RT or postoperative RT, is considered the gold standard surgery for the treatment of middle and low LARC. It is performed with precise dissection along an avascular, embryologically based plane, achieving good oncological and functional results (23, 32, 53). The supportive role of RT in patients treated with TME is challenged by, but not limited to, these three factors. First, low LR. Although several reliable clinic trials showed that RT significantly reduces LR by about 50% compared to TME alone, most of these trials were conducted more than 10 years ago (11, 12). Furthermore, those clinic trials conducted more than 10 years ago demonstrated that TME without RT alone results in a LR rate as low as about 10% (11, 12). Currently, mesorectal integrity is evaluated using high-resolution magnetic resonance imaging before surgery. When mesorectal integrity is used as the standard for evaluation, high-quality TME surgery results in a LR rate as low as 5% (32, 60). This raises the question of whether RT could further decrease the LR rate in patients treated with TME. An updated meta-analysis was conducted to evaluate the impact of RT on LR in LARC patients treated with TME. Data showed that neither preoperative nor postoperative RT significantly reduced LR. Second, there is a risk of RT-related complications, morbidities, and mortality (14, 26). RT can lead to complications such as enteritis, wound sepsis, anastomotic leak, among others. It has been reported that 90% of patients receiving pelvic RT experience chronic changes in their bowel habits, with half reporting a reduced quality of life attributable to these symptoms (61). RT-related complications not only cause a very negative impact on the quality of life but also increase the economic burden. Third, there is no observed benefit in OS. Survival is the most important endpoint in the treatment of rectal cancer. Data indicate that there was no significant difference in OS between the group receiving the combination of TME and RT and the TME-alone group. Further analysis indicates that neither preoperative RT nor postoperative RT benefits OS (Figure 8). Postoperative radiotherapy is not recommended for LARC by certain guidelines. However, it is not uncommon for a patient to be mis-diagnosed with early-stage rectal cancer before surgery, only to be diagnosed as LARC after surgery. Under this condition, postoperative radiotherapy is recommended by certain guidelines. Therefore, it may not be meaningless to discuss the role of postoperative radiotherapy. However, our results showed that postoperative radiotherapy did not improve the survival rate in patients with colorectal cancer, suggesting that caution should be exercised when considering its use. The HRs for preoperative RT, postoperative RT, and the combination of pre- and postoperative RT are all greater than one, indicating that the three RT groups had a shorter OS compared to the TME-alone group, although this difference was not statistically significant.

Therefore, preoperative RT or postoperative RT may not be necessary for all patients with completely resected LARC. RT might be cautiously recommended for a few patients at high risk. Factors such as mesorectal integrity, T stage, lymph node status, lesion location, among others, are considered in patient selection. Unfortunately, only a few studies have thoroughly explored the roles of these factors and further research is needed (31, 62, 63). Due to the high risk of surgery-related complications, morbidities, and mortality associated with TME—for example, an increased risk of anastomotic fistula with an incidence of about 11%–18% (64, 65)—a considerable number of patients undergo non-TME-only surgery (31, 66). Postoperative complications of colorectal cancer mainly include anastomotic leakage, bleeding, intestinal obstruction, surgical site infection, deep vein thrombosis, and other related conditions. These complications seriously affect the quality of life of patients, increase pain and length of hospital stay, and, in severe cases, can be life-threatening (67). Recently, it has been shown that changes in butyrylcholinesterase levels during colorectal surgery may be associated with the occurrence of complications, and monitoring these levels could potentially aid in the early prediction and intervention of postoperative complications (68). Currently, lateral lymph nodes are also considered potential sites of local lesions and should be managed with lateral lymph node dissection. Studies have shown that pelvic lymph node dissection guided by NIR fluorescence imaging is feasible. From the number of lymph nodes and postoperative outcomes, the technique demonstrates good performance, suggesting potential advantages in surgical efficacy and postoperative recovery. It is a technique worthy of attention (69). Data indicate that preoperative RT significantly benefits patients treated with non-TME-only surgery in terms of LR and OS (Figures 9, 10). Clinically, neoadjuvant chemoradiotherapy has become the standard treatment for LARC, achieving a high anal sphincter preservation rate. In some reports, this preservation rate can reach as high as 85.2%. However, the ultimate success of preserving the anal sphincter is also influenced by factors such as tumor location, stage, the patient’s physical condition, and other variables. According to the literature, further investigations have been conducted into the potential clinical application of deep learning algorithms for the classification and diagnosis of CRC histopathology images. The advancements made possible by deep learning algorithms have the potential to improve the accuracy and efficacy of CRC detection (70, 71). In recent decades, different RT regimens, including LCCRT and SCRT, have been applied to treat LARC. CRT tends to yield better survival outcomes than RT alone in certain types of cancers. A few trials have reported that LCCRT yielded a higher rate of pCR, lower LR rates, and reduced T and N stages compared to SCRT (72, 73). The impact of different RT regimens on OS was analyzed. Preoperative RT alone, preoperative CRT, preoperative LCCRT, and preoperative SCRT all benefit patients treated with non-TME-only surgery in terms of OS (Figure 10). Therefore, preoperative RT is essential for patients with completely resected middle and low LARC. The data here show that postoperative RT did not improve OS (Figure 4). This suggests that postoperative RT may not be necessary for all patients with completely resected LARC. Postoperative RT may be cautiously recommended for selected high-risk patients.

Over two decades ago, meta-analyses showed that postoperative RT had no impact on survival, while preoperative RT had a significant positive effect (74, 75). At present, modern precision radiotherapy techniques, such as 3-dimensional conformal radiotherapy (3D-CRT) and intensity-modulated radiotherapy (IMRT), are widely used. These techniques allow for the delivery of high radiation doses to the gross tumor, bulky lymph nodes, and high-risk areas, while minimizing the dose and preserving the volume of organs at risk (76). Nevertheless, the conclusion of this updated meta-analysis remains unchanged. Postoperative RT does not benefit patients in terms of OS, while preoperative RT significantly improves OS (Figures 4–6). Further analysis suggests that the effect of preoperative RT may be attributable to its impact on patients who underwent non-TME-only surgery, rather than those who received TME.

In addition to the inherent limitations of individual trials, there are also limitations to our analyses. First, treatment modalities vary considerably among different clinic trials. Different chemotherapy regimens were employed in different clinic trials, and even within a single trial. Some patients received chemoradiotherapy, while others received RT alone. Additionally, different RT techniques were used. These factors pose a significant risk of bias in the implementation of the meta-analysis. Second, patients with different T-stages and different lymph node statuses were included. The tumor’s biological heterogeneity and perineural and vascular invasion in different patients were not considered. Different patients have varying risks of LR, and RT might benefit patients with different risks profiles in distinct ways. These confounders affect the efficacy of RT more or less. Third, the sample sizes in different trials vary considerably. Without considering the impact of differences in TME resection techniques on surgical outcomes and patient prognosis, variations in the TME techniques employed by different operators may result in different margin statuses, LR rates, and other outcomes. Fourth, some database, such as Web of Science and others, were not included in the search due to limited access. Because of limitations, data should be interpreted with caution. Clinicians should carefully evaluate the indications to ensure favorable oncological outcomes and inform patients about the potential risks to functional outcomes.

## 5 Conclusion

Preoperative RT, regardless of the regimen, benefits LARC patients treated with non-TME-only surgery in terms of OS, while preoperative RT does not improve OS in patients treated with TME. Postoperative RT does not improve OS in patients with completely resected LARC. Due to these limitations, data should be interpreted with caution.

## Abbreviations

(TME), total mesorectal excision; (non-TME-only), other types of surgery; (OS), overall survival; (LCCRT), long-course concurrent chemoradiotherapy; (RT), radiotherapy; (MDT), multidisciplinary treatment; (LARC), locally advanced rectal cancer; (LR), local recurrence; (CRT), concurrent chemoradiotherapy; (DFS), disease-free survival; (DMFS), distant metastases-free survival; (HR), hazard ratio; (OR), odds ratio; (3D-CRT), 3-dimensional conformal radiotherapy; (IMRT), intensity-modulated radiotherapy; (pCR), pathological complete response; (TNT), total neoadjuvant therapy.
