# Supplementary material for: Efficacies of radiotherapy in rectal cancer patients treated with total mesorectal excision or other types of surgery: an updated meta-analysis
Source: Oncol Rev. 2025 May 1;19:1567818. doi: 10.3389/or.2025.1567818 (PMC12078337; doi:10.3389/or.2025.1567818)
Supplement: Supplementary file 1 [file DataSheet1.docx]

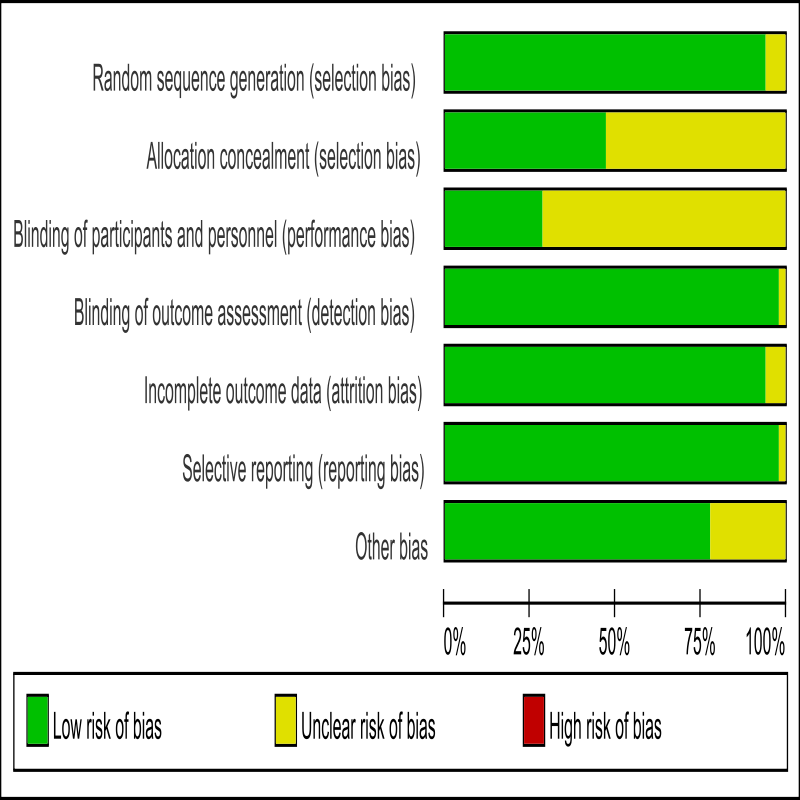


Figure 1. Risk of bias graph.


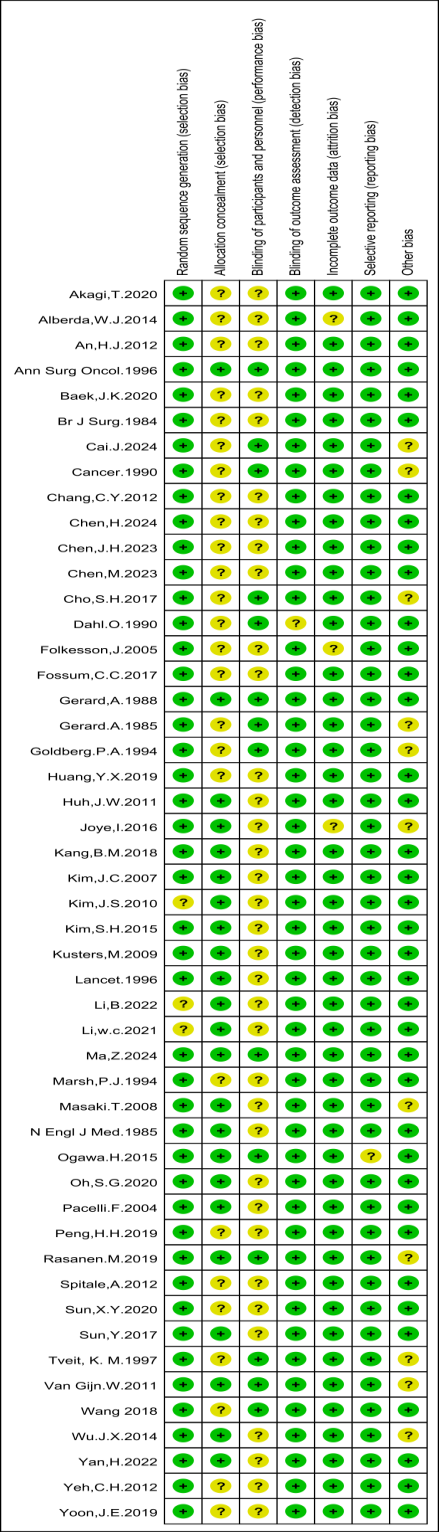


Figure 2. Risk of bias summary.


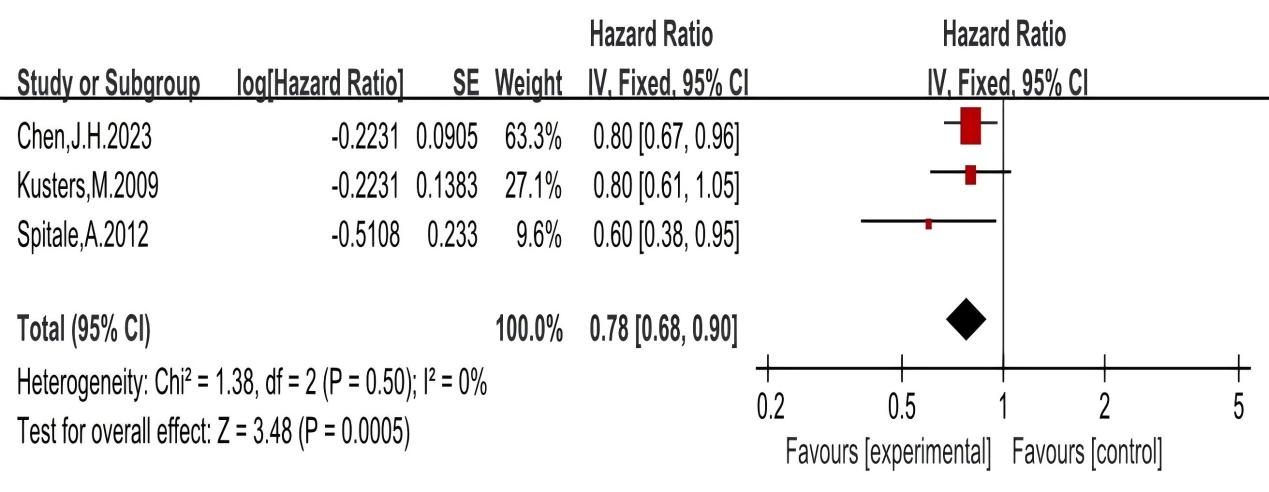


Figure 3. Pooled HRs for cancer-specific survival in patients treated with RT.


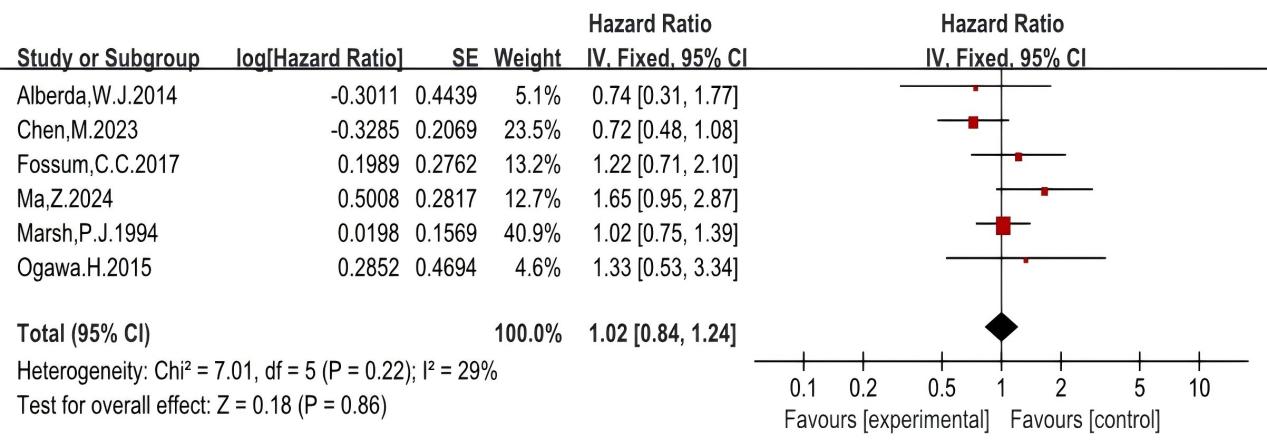


Figure 4. Pooled HRs for metastasis-free survival in patients treated with RT.


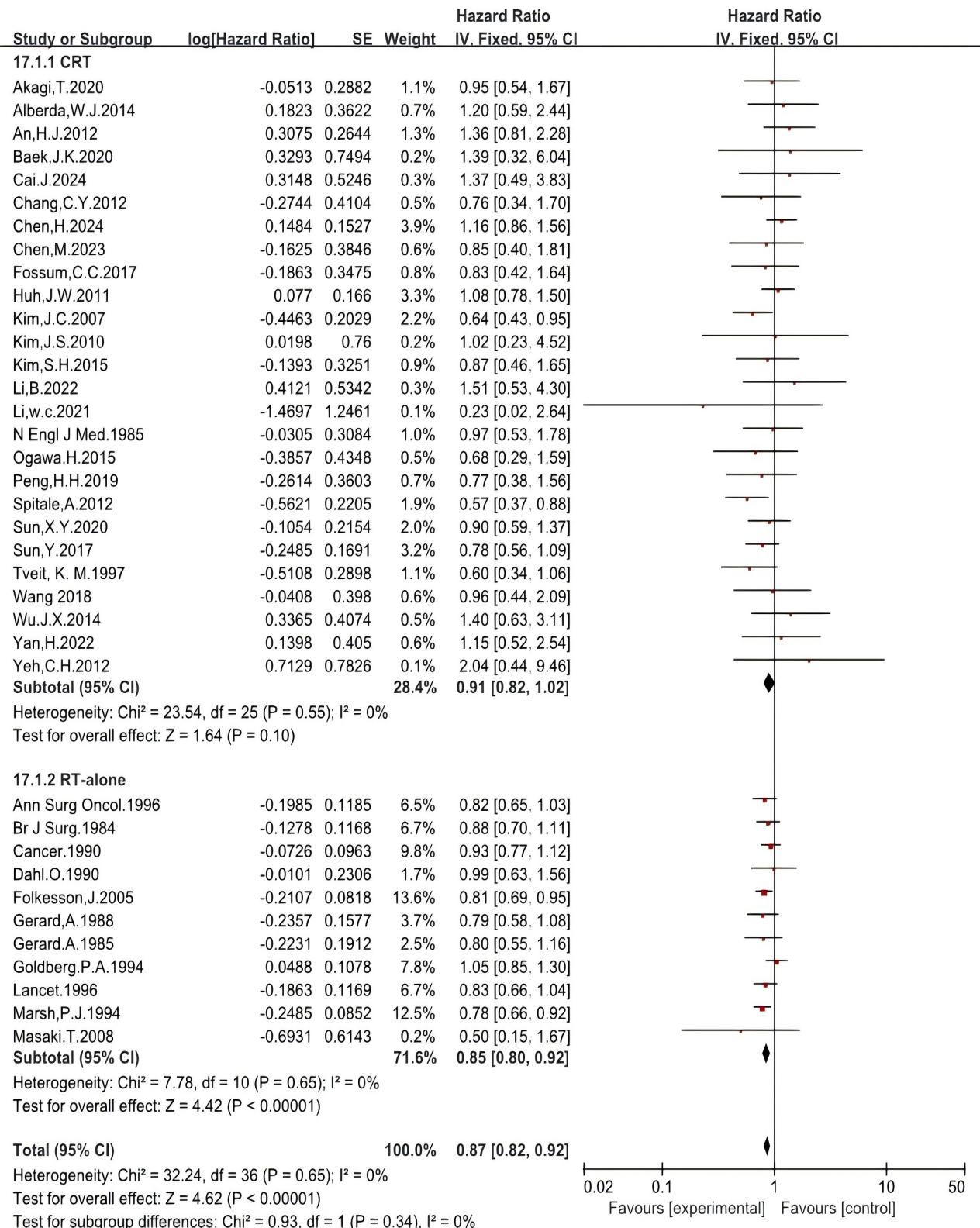


Figure 5. Impacts of CRT and RT-alone on OS.


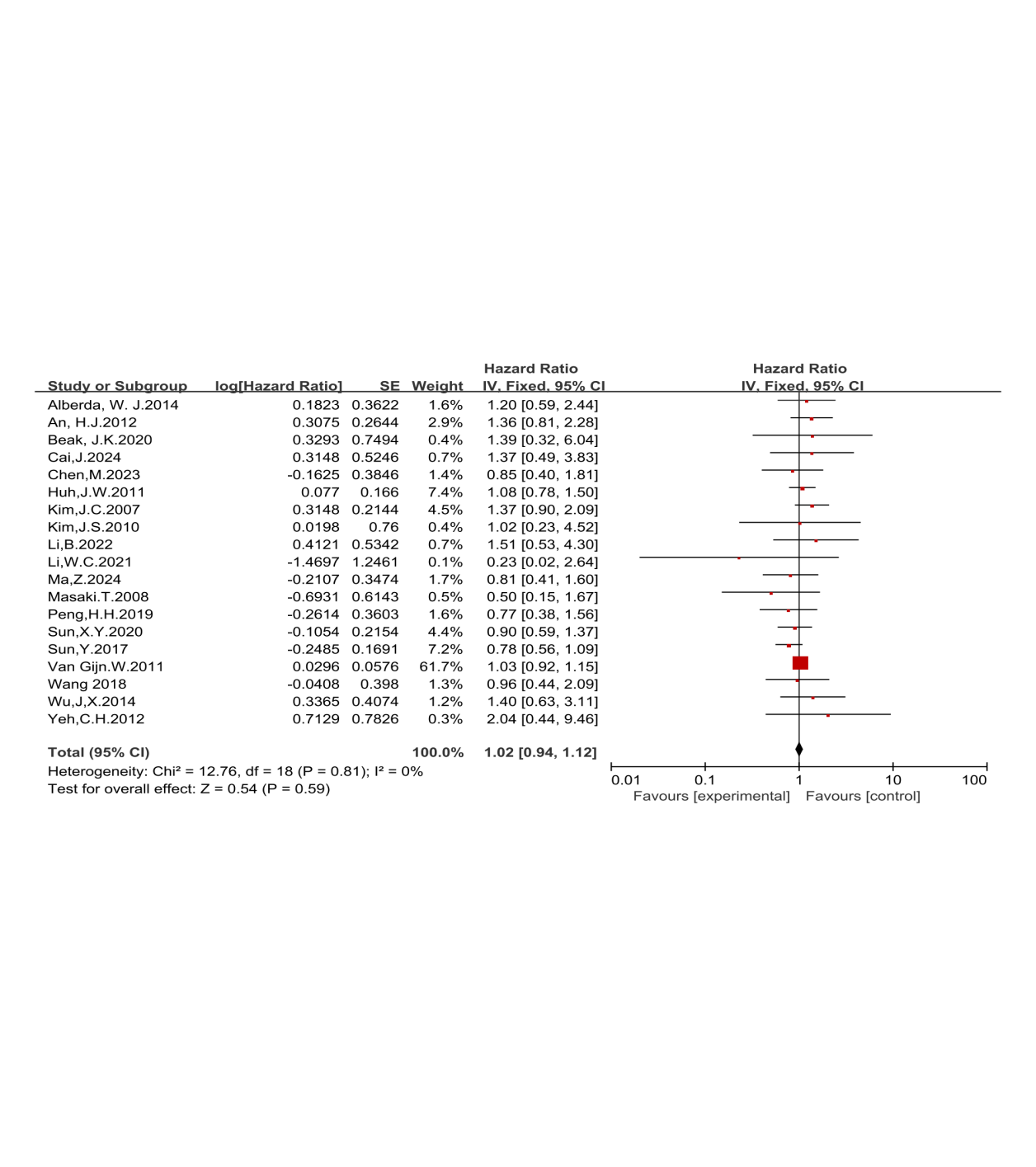


Figure 6. Impact of RT on OS in patients treated with TME.


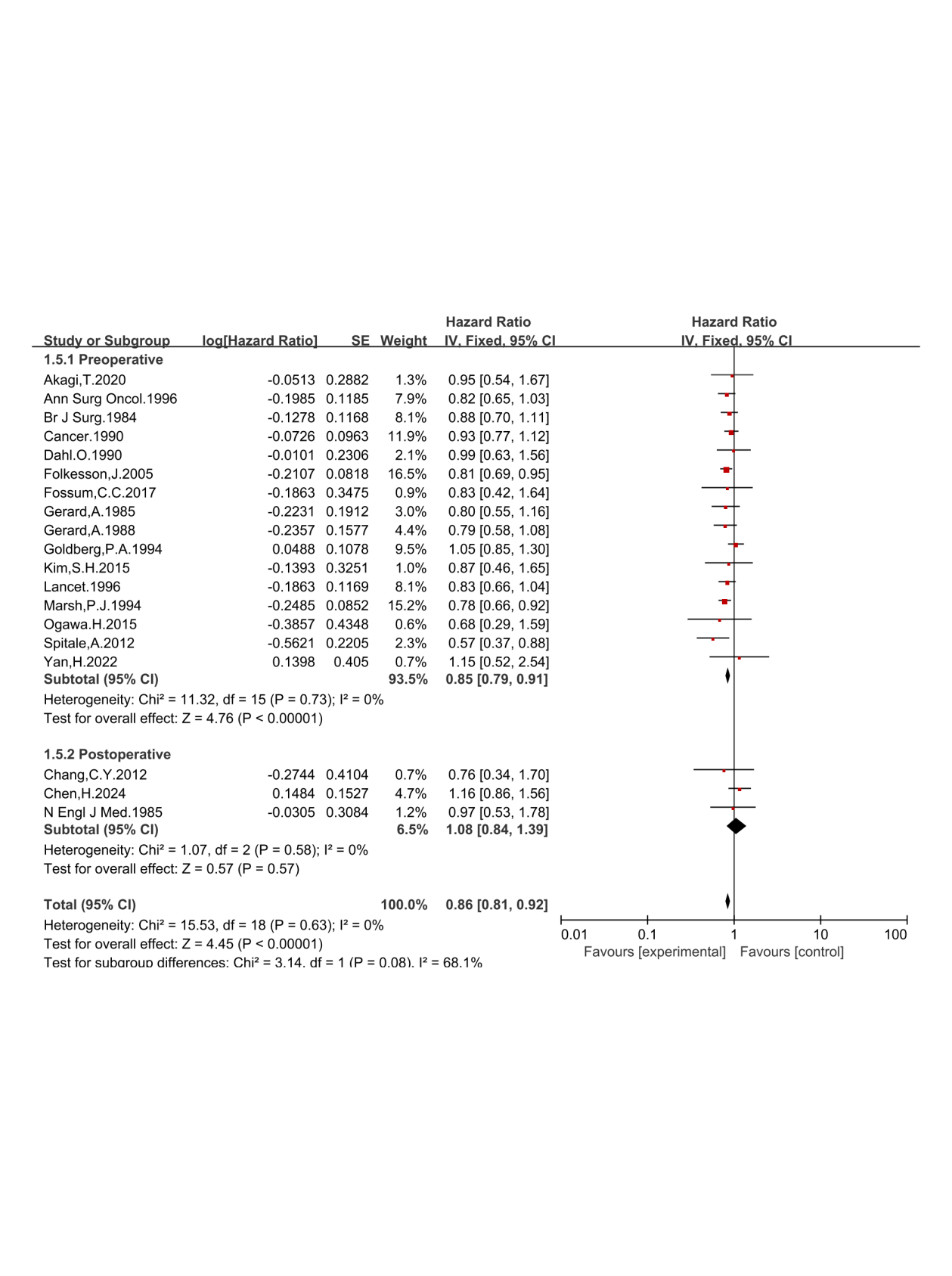


Figure 7. Impacts of preoperative RT and postoperative RT on OS in patients treated with non-TME sugery.

**Literature research**

The search strategy for PubMed or Cochrane Library is as followed: (radiation or irradiation or RT or chemoradiation or chemoradiotherapy or radiochemotherapy) and (rectal cancer or rectal carcinoma) in (Abstract or Title). Search strategy for CNKI is as followed: (放疗 or 放射治疗) and (直肠癌) in (Abstract or Title)
